# A novel DNA damage response mediated by DNA mismatch repair in *Caenorhabditis elegans*: induction of programmed autophagic cell death in non-dividing cells

**DOI:** 10.18632/genesandcancer.70

**Published:** 2015-07

**Authors:** Takahito Moriwaki, Yuichi Kato, Chihiro Nakamura, Satoru Ishikawa, Qiu-Mei Zhang-Akiyama

**Affiliations:** ^1^ Laboratory of Stress Response Biology, Graduate School of Science, Kyoto University, Kitashirakawa-Oiwakecho, Sakyo-ku, Kyoto, Japan

**Keywords:** MLH1, ATM, ATR, Organism death, Cancer

## Abstract

DNA mismatch repair (MMR) contributes to genome integrity by correcting errors of DNA polymerase and inducing cell death in response to DNA damage. Dysfunction of MMR results in increased mutation frequency and cancer risk. Clinical researches revealed that MMR abnormalities induce cancers of non-dividing tissues, such as kidney and liver. However, how MMR suppresses cancer in non-dividing tissues is not understood.

To address that mechanism, we analyzed the roles of MMR in non-dividing cells using *Caenorhabditis elegans* (*C. elegans*), in which all somatic cells are non-dividing in the adult stage. In this study, we used stable MMR-mutant lines with a balancer chromosome. First, we confirmed that deficiency of MMR leads to resistance to various mutagens in *C. elegans* dividing cells. Next, we performed drug resistance assays, and found that MMR-deficient adult worms were resistant to SN1-type alkylating and oxidizing agents. In addition, dead cell staining and reporter assays of an autophagy-related gene demonstrated that the cell death was autophagic cell death. Interestingly, this autophagic cell death was not suppressed by caffeine, implying that MMR induces death of non-dividing cells in an *atl-1*-independent manner. Hence, we propose the hypothesis that MMR prevents cancers in non-dividing tissues by directly inducing cell death.

## INTRODUCTION

DNA mismatch repair (MMR) ensures genome integrity by correcting DNA replication and recombination errors [1]. MMR is highly conserved from bacteria to humans [2,3]. In eukaryotes, MMR starts with detection of mismatches by MSH2-MSH6 heterodimer (MutSα) followed by interacting with MLH1-PMS2 heterodimer (MutLα), proliferating cell nuclear antigen (PCNA), and exonuclease1 (EXO1), and then that complex excises the newly synthesized strand by exonuclease activity of EXO1[1]. Dysfunction in MMR results in an increased mutation frequency in many kinds of organisms [4]. MMR abnormalities in humans result in increased risks of cancer, including hereditary nonpolyposis colorectal cancer (HNPCC) [5]. It is considered that MMR-deficient colorectal cells rapidly accumulate mutations due to their high turnover, which ultimately induces carcinogenesis. On the other hand, it has been reported that dysfunction of MMR also leads to increased cancer risks in non-dividing tissues, such as kidney and liver [6,7]. However, it is not understood how MMR suppresses carcinogenesis in non-dividing cells.

Recent studies have revealed that MMR plays key roles in the DNA damage response (DDR) process [8]. An *in vitro* analysis revealed that MutSα has affinity to O^6^-methylguanine, which is generated by SN1-type DNA alkylation [9]. Furthermore, an *in vivo* analysis using mammalian cultured cells showed that following recognition of the DNA lesions, MMR activates the G2/M checkpoint or apoptosis pathway via the ataxia telangiectasia and Rad3-related protein (ATR)/ATR Interacting Protein (ATRIP) pathway after forming a complex with MutLα, PCNA, and EXO1 [10–12]. Due to the lack of that apoptotic pathway, MMR-deficient cells show resistance to SN1-type alkylating agents [13]. In addition to O^6^-methylguanine, a wide range of DNA lesions have been identified as substrates of MMR, such as apurinic/apyrimidinic (AP) sites, interstrand cross-links, 8-oxoguanine, and UV-photoproducts [14–17]. Although some reports have shown the possibility that MMR can also function in non-dividing cells, for example, by noncanonical MMR, the physiological function thereof is not well understood [18,19].

In order to address the relationship between the dysfunction of MMR and carcinogenesis in non-dividing cells, we investigated the roles of MMR in non-dividing cells using the nematode *Caenorhabditis elegans* (*C. elegans*). *C. elegans* has been extensively used as a model organism for aging, the nervous system, and apoptosis. The cell fates of every cell in *C. elegans* have been completely determined, and all the somatic cells of an adult worm are non-dividing [20,21]. Also in *C. elegans*, dysfunction of the MMR gene results in increased mutation frequency; for instance, knockdown of *mlh-1*, the homologue of *hMlh1*, results in increased mutation frequency [22].

Recently, it was reported that in response to 5-fluorouracil (5-FU) treatment, MMR induces expression of *lgg-1* (*C. elegans MAP1LC3* orthologue), one of the autophagosome genes in a manner dependent on ATL-1 (*C. elegans* ATR homologue) and two AP-endonucleases, EXO-3 and APN-1 in *C. elegans* embryos [23]. 5-FU treatment inhibits thymidine synthetase and incorporation of 5-fluorodeoxyuridine in genomic DNA [24]. In that pathway, MMR induces autophagic cell death in response to 5-FU treatment in embryos, indicating that *C. elegans* MMR recognizes 5-fluorodeoxyuridine in genomic DNA and induces autophagic cell death. However, other possible substrates that could similarly induce autophagy have not been identified yet in *C. elegans*. In the present study, we performed drug treatments of N2 and *mlh-1(ok1917)* mutant worms at various developmental stages. *mlh-1(ok1917)* worms have been used previously for analysis of the relationship between MMR and other DNA repair pathways [25,26]. We first performed screenings of substrates of *C. elegans* MMR using pachytene stage meiotic cells, in which apoptosis is induced in response to DNA damage [27]. The results revealed that *mlh-1(ok1917)* pachytene stage meiotic cells showed decreased apoptotic cells in response to various DNA lesions, like mammalian cultured cells. In addition, we confirmed by L1 drug resistance assays that MMR induces cell cycle arrest or cell death in response to DNA damage in dividing somatic cells. We next performed drug resistance assays using adult worms, and found that *mlh-1(ok1917)* adult worms showed resistance to alkylating and oxidizing agents. In addition, the results of reporter assays of *lgg-1*, one of the autophagosome genes, suggested that the cell death of non-dividing adult somatic cells was not apoptosis but autophagic cell death. Interestingly, this autophagic cell death was not suppressed by caffeine treatment, suggesting that autophagic cell death is independent on ATL-1. These results clearly demonstrate that *C. elegans* MMR can induce cell death in non-dividing cells in a manner apparently different from that in embryos, and suggest that MMR prevents carcinogenesis in non-dividing tissues by inducing cell death.

## RESULTS

### Establishment of a stable MMR mutant worm line

Previous studies showed that dysfunction of MMR results in increased mutation frequency in *C. elegans* [22]. Backcrossed *msh-2*(*ev679*::*Tc1*) worms have no abnormalities in basic phenotypes, such as growth and lifespan. However, they die out during serial passage in culture, because of accumulation of mutations [28]. These facts mean that a stable maintenance system for MMR mutants is required to analyze the function of MMR in DDR without disturbing background mutations. In previous studies, it was shown that an increased mutation frequency is not observed in MMR heterozygous mutants in various species [29,30]. Therefore, we first established a stable maintenance system for the MMR heterozygous mutant using a balancer chromosome, *hT2* (Supplementary Figure S1). *hT2* is one of the balancer chromosomes that prevents parts of chromosomes I and III from homologous recombination. In addition, the *myo-2*p::GFP gene and recessive lethal gene were inserted as markers, and we could maintain the heterozygous mutants by selective picking of GFP-positive worms [31].

*C. elegans* MLH-1 is a component of *C. elegans* MutLα, and knockdown of *C. elegans mlh-1* results in increased mutation frequency [22]. We performed backcrossing of the *mlh-1*-deficient strain RB1572 (*mlh-1(ok1917)*), with N2 worms (wild-type). After backcrossing, we crossed the worms with JK2739 (*lin-6*(*e1466*) *dpy-5*(*e61*) *I/hT2* [*bli-4*(*e937*) *let-?*(*q782*) *qIs48*] (*I;III*)) worms to maintain the MMR mutant line in genetically stable condition (*mlh-1*(*ok1917*/*hT2*)). We maintained *mlh-1*(*ok1917*/*hT2*) worms by picking GFP-positive worms until use and isolated *mlh-1*(*ok1917*/*ok1917*) worms for experiments by picking GFP-negative worms (Supplementary Figure S1). We confirmed the absence of abnormalities in basic phenotypes in the backcrossed *mlh-1(ok1917)* mutant line (Supplementary Figure S2).

### *mlh-1(ok1917)* pachytene stage meiotic cells showed decreased apoptosis in response to a wide range of DNA-damaging agents

In previous research, it was reported that MMR induces autophagic cell death in response to 5-FU treatment in a manner dependent on two AP endonucleases, EXO-3 and APN1, in *C. elegans* embryos [23]. 5-FU induces genome instability by incorporation of 5-fluorodeoxyuridine into genomic DNA during DNA replication [32]. Therefore, 5-FU is not suitable for our research focused on the function of MMR in the DNA damage response in non-dividing cells. In order to identify other possible substrates, we measured the number of apoptotic pachytene stage meiotic cells. In *C. elegans*, apoptosis in pachytene stage meiotic cells is frequently induced by DNA damage and causes the formation of condensed structures in the gonad that are easily detected by DIC microscopy [33]. We treated synchronized adult worms for 24 hours with various drugs, and measured the number of apoptotic cell corpses of pachytene stage meiotic cells.

**Figure 1:**
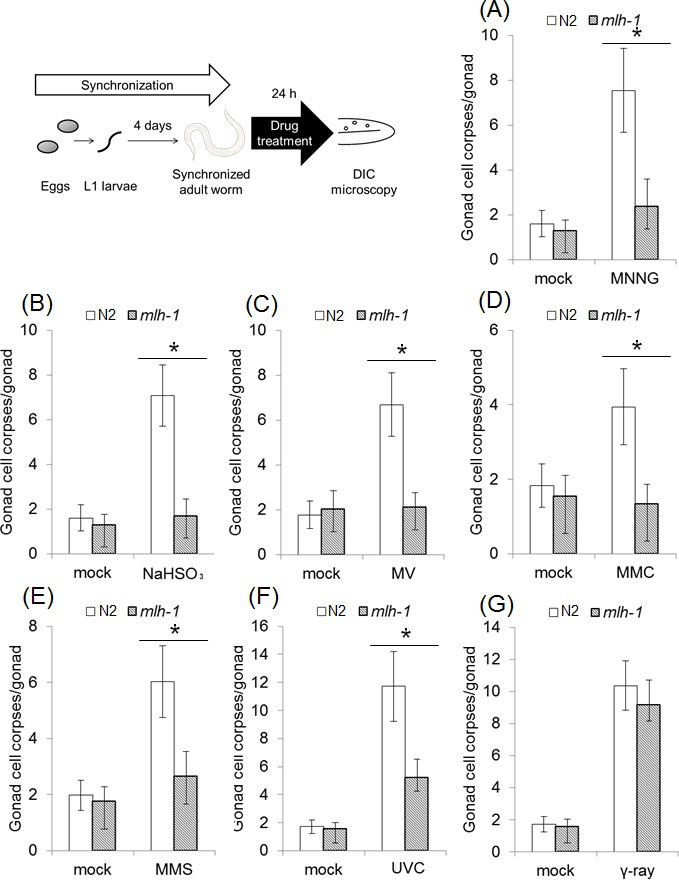
MLH-1 deficiency leads to resistance to various mutagens in pachytene stage meiotic cells (A-E) The number of pachytene stage meiotic cells' death of N2 and *mlh-1(ok1917)*. The number of pachytene stage meiotic cells' death was measured following 24-hour drug treatment. Adult worms were treated with **(A)** 0.25 mM MNNG, **(B)** 25 mM NaHSO_3_, **(C)** 10 mM MV, **(D)** 0.2 mg/ml MMC, or **(E)** 0.05% MMS for 24 hours at 20°C. After the treatments, dead pachytene stage meiotic cells were counted by DIC microscopy. (F and G) The number of germline corpses after 24 hours of irradiation. Adult worms were irradiated with **(F)** 150 J/m^2^ UVC or **(G)** 100 Gy γ-rays. Germ cell corpses were counted by DIC microscopy 24 hours after irradiation. All data represent the mean ± 95% confidence interval from three independent experiments. * indicates *p*-value<0.05 by the Mann-Whitney U-test. A photograph of a worm was obtained from the Togo picture gallery (http://g86.dbcls.jp/~togoriv/). ©2011 DBCLS Licensed under a Creative Commons Attribution 2.1 Japan License.

N-methyl-N'-nitro-N-nitrosoguanidine (MNNG) is an SN1-type alkylating agent that is frequently used in research on MMR-dependent cell death and cell cycle arrest. MNNG mainly generates O^6^-methylguanine via SN1-type alkylation in genomic DNA [9]. MNNG treatment increased the number of apoptotic pachytene stage meiotic cells in N2 gonad arms, but such an increase was not observed in the *mlh-1(ok1917)* gonad arms (Figure 1A). These results indicate that *C. elegans* MMR induces apoptosis in response to MNNG treatment, as it does in mammalian cultured cells.

In addition, we also tested the following types of DNA-damaging agents: SN2-type alkylating agent, deaminating agent, oxidizing agent, cross-linking agent, UVC, and γ-rays. Methyl methanesulfonate (MMS) is an SN2-type alkylating agent, and in contrast to SN1-type alkylating agents mainly generates unstable DNA lesions such as 7-methylguanine and 3-methyladenine, which are easily converted to AP sites [34]. Mitomycin C (MMC) is a cross-linking agent that generates interstrand cross linking in genomic DNA [35]. Methyl viologen (MV), which is also called paraquat, is a superoxide-inducing agent, and generates oxidative DNA lesions in genomic DNA [36,37]. Sodium hydrogen sulfite (NaHSO_3_) is a deaminating agent that generates deaminated DNA lesions such as uracil and 5-(hydroxymethyl) uracil in genomic DNA [38]. UVC irradiation induces dimerization of pyrimidine bases [39]. These DNA-damaging agents have been reported to be activators of MMR in previous studies [9,14–17]. Pachytene stage meiotic cells of *mlh-1(ok1917)* showed less apoptosis than those of N2 worms in response to all of the types of DNA-damaging treatments which we tested, except γ-rays irradiation (Figures 1B-F). These results suggest that *C. elegans* MMR can induce cell death in response to various base modifications. However, γ-rays irradiation induced a similar increase of death of pachytene stage meiotic cells in N2 as in *mlh-1(ok1917)* (Figure 1G). This is considered to have been due to the cytotoxicity of DNA double-strand breaks (DSBs) induced by γ-rays.

### *C. elegans* MMR regulated larval development

**Figure 2:**
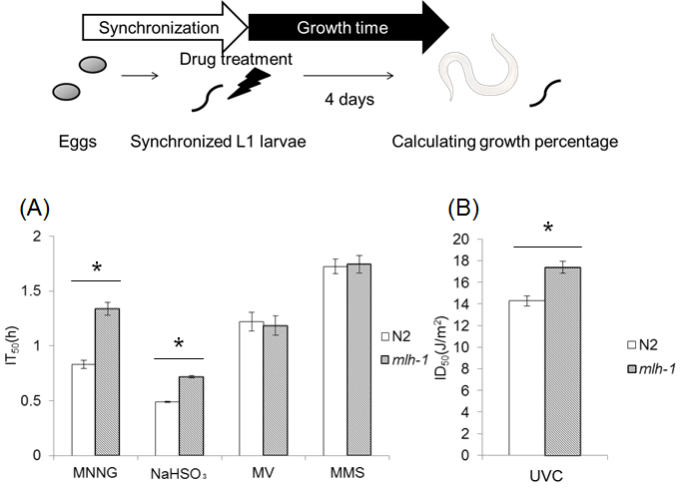
mlh-1(ok1917) worms showed increased percent growth under mutagen treatment The synchronized starved L1 larvae of N2 and *mlh-1(ok1917)* were treated with **(A)** 0.6 mM MNNG, 60 mM NaHSO, 40 mM MV, or 0.05% MMS and **(B)** irradiated with UVC. After the treatments or irradiation, worms were cultured for 4 days on NGM plates at 20°C. We then calculated IT_50_ or ID_50_ from the normalized percent growth (L1 to L4). Error bars show 95% confidence interval. * indicates *p*-value<0.05 by Student's *t*-test. A photograph of a worm was obtained from the Togo picture gallery (http://g86.dbcls.jp/~togoriv/). ©2011 DBCLS Licensed under a Creative Commons Attribution 2.1 Japan License.

Microarray experiments revealed that *C. elegans* MMR genes *msh-2*, *msh-6*, *mlh-1* and *pms-2* are abundantly expressed in pachytene stage meiotic cells and embryos, and their expression levels are decreased after hatching by approximately 10-fold, except for that of *pms-2* [40,41]. Next, we examined whether *C. elegans* MMR induces cell death or cell cycle arrest in post-embryonic cells, dividing somatic cells (dSCs), using L1 larvae. *C. elegans* larvae develop to adult worms through 4 stages or molts, which are designated as L1, L2, L3 and L4. We synchronized worms at L1 stage and performed a time-course of treatment with various DNA-damaging agents. Four days after the drug treatments, we measured the growth (percentage) from L1 larvae to the L4-adult stages, and calculated the half maximal inhibitory time (IT_50_) or dose (ID_50_) from three-parameter logistic curves (Supplementary Figure S3). *mlh-1(ok1917)* larvae showed significantly higher IT_50_ or ID_50_ in response to MNNG, NaHSO, and UVC than N2 larvae (Figures 2A and B). This suggests that *C. elegans* MMR induces cell cycle arrest or cell death in dSCs. In contrast, no significant difference was observed in the sensitivity to MV or MMS between N2 and *mlh-1(ok1917)* (Figure 2A). Such a difference was observed for the *msh-6*(*pk2504*) mutant (Supplementary Figures S4A and B). The observed difference of response suggests that there may be differential responses to DNA damage between pachytene stage meiotic cells and proliferating larval cells, although this difference may just be due to differences of drug penetrance or replication stress. In addition, we tested MMC and γ-rays, and found that they did not affect percentage growth significantly at the maximum doses used, 0.2 mg/ml and 100 Gy, respectively, in either N2 or *mlh-1(ok1917)* L1 larvae (data not shown).

### *C. elegans* MMR induced non-dividing cell death in response to DNA damage

**Figure 3:**
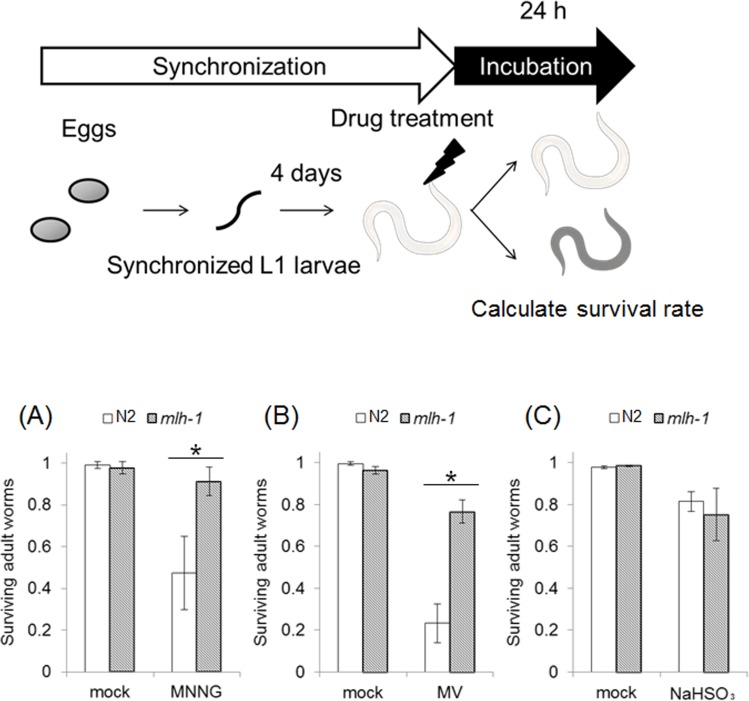
MMR induced organism death independent of DNA replication The adult worms of N2 and *mlh-1(ok1917)* were treated with **(A)** 3.4 mM MNNG, **(B)** 200 mM MV, or **(C)** 0.1 M NaHSO_3_ for 1 hour at 20°C. After the treatment, worms were cultured for 24 hours at 20°C and the survival rate was calculated. All data represent the mean ± S.D. from three independent experiments. * indicates *p*-value<0.05 by Student's *t*-test. A photograph of a worm was obtained from the Togo picture gallery (http://g86.dbcls.jp/~togoriv/). ©2011 DBCLS Licensed under a Creative Commons Attribution 2.1 Japan License.

We confirmed that *C. elegans* MMR induces cell death or cell cycle arrest in post embryonic cells, dSCs, in response to some mutagens, including MNNG. Next, we examined the drug resistance of non-dividing somatic cells (ndSCs) using adult worms. We prepared fully matured adult worms by culturing synchronized L1 larvae on NGM plates for 4 days, and treated them with DNA-damaging agents for 1 hour. 24 hours after the drug treatments, we measured survival rate. As shown in Figures 3A and B, *mlh-1(ok1917)* adult worms showed a significantly higher survival rate than N2 worms after the MNNG and MV treatments. Moreover, no significant difference of the sensitivity to NaHSO_3_ treatment was observed (Figure 3C).

**Figure 4:**
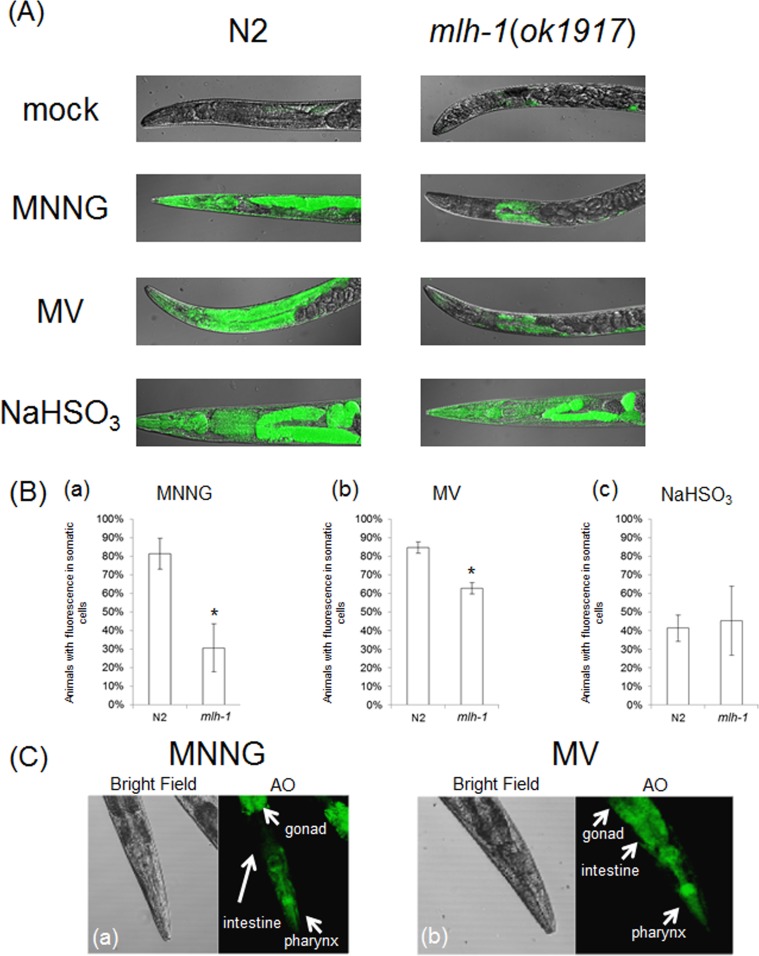
MMR induced tissue-specific cell death **(A)** MLH-1 deficiency suppressed cell death. The adult worms of N2 and *mlh-1(ok1917)* were non-treated or treated with 3.4 mM MNNG, 200 mM MV or 0.1 M NaHSO_3_ for 1 hour at 20°C. After the treatment, worms were stained with AO for 5 minutes at 20°C and observed microscopically after destaining two times with M9 buffer and fixing with PFA. **(B)** The fraction of animals with somatic AO fluorescence. All data represent the mean ± S.D. from three independent experiments. * indicates *p*-value<0.05 by Student's *t*-test. **(C)** A more highly magnified image of AO-stained N2 worms. White arrows indicate the location of each organ.

In order to address whether *C. elegans* MMR induces ndSCs' death, we performed acridine orange (AO) staining. AO is a non-fluorescent dye that shows fluorescence when it binds stably to nucleic acids. Because AO is actively exported out of living cells, fluorescence is observed only in dead cells after sufficient destaining [42]. As shown in Figures 4A and B, there were significantly fewer worms with AO fluorescence in *mlh-1(ok1917)* than in N2 in the cases of MNNG and MV treatment. In contrast, the same levels of AO fluorescence were observed in the case of NaHSO_3_ treatment (Figures 4A and B). Moreover, AO-induced fluorescence was especially observed in the pharynx and intestines of N2 worms after MV treatment, whereas there was no such localization of the fluorescence of *mlh-1(ok1917)* worms (Figure 4A). As shown in Figure 4C, MNNG treatment induced not only pharynx cell death but also death of the surrounding cells, while MV treatment induced only pharynx cell death in the head part. In *C. elegans*, the pharynx is in the head part, and neurons are located surrounding the pharynx. These results suggest that MNNG and MV especially injure neurons and intestine, respectively, in an MMR-dependent manner in *C. elegans*.

### *C. elegans* MMR induced autophagic cell death in ndSCs independently of ATL-1

In humans, MMR induces apoptosis or cell cycle arrest via the ATR/ATRIP pathway [11]. In response to damage recognition by MutSα, ATR/ATRIP is activated and phosphorylates CHK1 to induce cell cycle arrest and CHK2 to induce apoptosis [11,12]. In *C. elegans*, this system is well conserved, and it has been reported that in *C. elegans* embryos, MMR activates CHK-1 (*C. elegans* CHK1 homologue) and induces autophagic cell death in response to 5-FU treatment in an ATL-1-dependent manner [23]. In order to examine how MMR induces cell death in ndSCs, we next tried to identify the type of cell death. In *C. elegans*, the cascade of apoptosis is well understood, and is regulated by only 4 factors: CED-3, CED-4, CED-9, and EGL-1. CED-3 is the effector caspase, and is converted to the active form by CED-4. Usually, CED-4 is blocked by CED-9, but only when cells undergo apoptosis, CED-4 activates CED-3 as a result of inhibition of CED-9 by EGL-1. *egl-1* is one of the genes whose expression is regulated by CEP-1 (the *C. elegans* p53 homologue) and is responsible for apoptosis in pachytene stage meiotic cells [43]. To determine whether the cell deaths in ndSCs were due to apoptosis, we measured somatic *egl-1* mRNA levels in MNNG-treated worms by semi-quantitative RT-PCR using the temperature-sensitive sterile mutant *glp-4*(*bn2*) cultured at 25°C, which does not have gonads, but no significant induction of *egl-1* mRNA was detected, whereas Wt worms, which have gonads, showed increased *egl-1* mRNA in response to MNNG treatment (Supplementary Figure S5).

**Figure 5:**
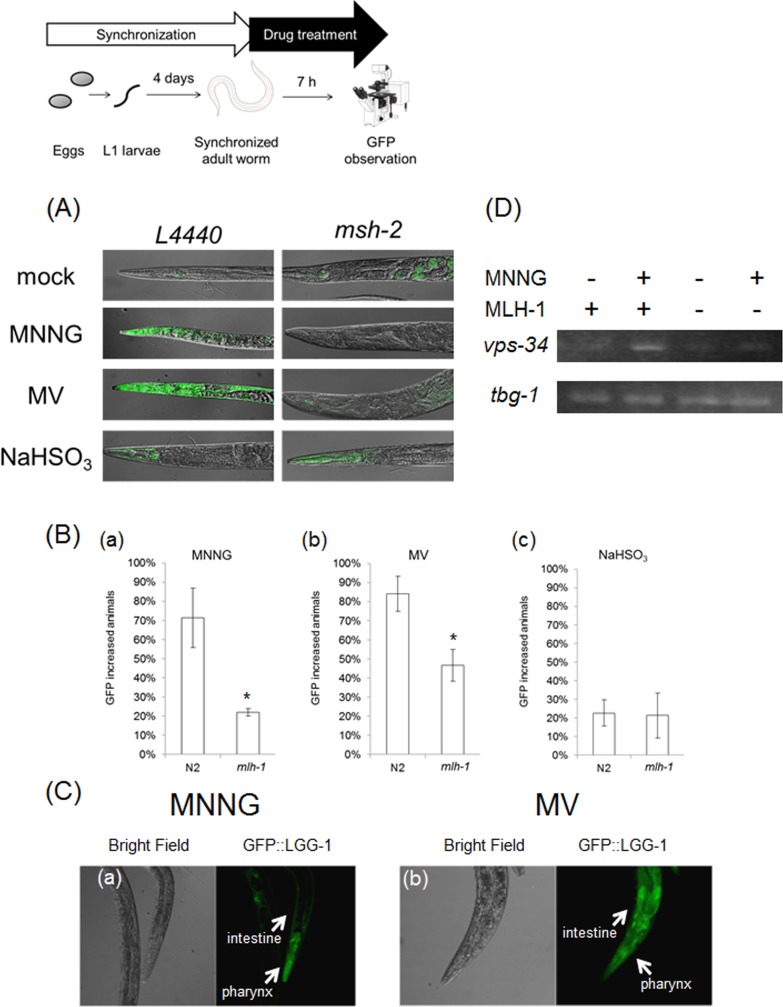
MMR induced autophagic cell death **(A)** Reporter assays of *lgg-1*. Synchronized MAH236 (*lgg-1*p::GFP::*lgg-1*) adult worms cultured on control or RNAi plates were treated with 1.7 mM MNNG, 50 mM MV or 50 mM NaHSO_3_ for 7 hours at 20°C. After the treatments, worms were fixed with PFA. After washing two times with M9 buffer, worms were examined microscopically. **(B)** The fraction of animals with increased somatic GFP. All data represent the mean ± S.D. from three independent experiments. * indicates *p*-value<0.05 by Student's *t*-test. At least 150 animals were observed in each condition. **(C)** Enlarged images of (a) MNNG-treated and (b) MV-treated MAH236 worms cultured on control plates. **(D)** Semi-quantitative RT-PCR of MNNG-treated worms. The adult worms of wild-type or *ok1917* germline deficient worms (*glp-4*(*bn2*)) were treated with 1.7 mM MNNG for 7 hour at 20°C. Then, total RNA was purified and reverse transcribed. The expression levels of *vps-34* were determined by semi-quantitative RT-PCR. *tbg-1* was used as the loading control. A photograph of a worm and microscopic images were obtained from the Togo picture gallery (http://g86.dbcls.jp/~togoriv/). ©2011 DBCLS Licensed under a Creative Commons Attribution 2.1 Japan License.

We then focused on autophagic cell death. Autophagic cell death is also known as type 2 cell death, and is defined as cell death with autophagy rather than apoptosis [44,45]. In humans, MAP1LC3 (ATG8) is widely used as a marker of autophagic cell death. In mammalian cells, MAP1LC3 exists in two forms: full-length form MAP1LC3-I (MAP1LC3) and shorter-form MAP1LC3-II [45]. During autophagy, MAP1LC3-I is transformed to MAP1LC3-II by cleavage, and MAP1LC3-II localizes to membranes of autophagosomes [45]. In the process of autophagic cell death, *MAP1LC3* is specifically upregulated [44,45]. This system is well conserved also in *C. elegans*, and upregulation of *lgg-1* in the process of autophagic cell death has been reported [23]. Based on these facts, we used *lgg-1* expression as a marker of autophagic cell death, and we performed reporter assays using MAH236 (*lgg-1*p::GFP::*lgg-1*) worms. Because of the expressed GFP::LGG-1, we could not use a stable maintenance system using a GFP balancer. Therefore, we performed reporter assays using *msh-2* knockdown worms (Supplementary Figure S6). As shown in Figures 5A and B, the expression level of *lgg-1* was elevated by MNNG and MV treatments, and knockdown of *msh-2* suppressed this induction, whereas there was no difference between the NaHSO-treated control and *msh-2* knockdown worms. LGG-1 was expressed in the pharynx and intestine in MV-treated N2 worms, but only in the pharynx and surrounding cells in MNNG-treated N2 worms (Figure 5C). In addition, we analyzed the mRNA levels of *vps-34*, a homologue of human class III PI3K, which is expressed during autophagic cell death in *C. elegans*, and we confirmed that mRNA levels of *vps-34* were increased in response to MNNG treatment (Figure 5D) [23]. These results suggest that MMR induces autophagic cell death in ndSCs in response to MNNG and MV treatment.

**Figure 6:**
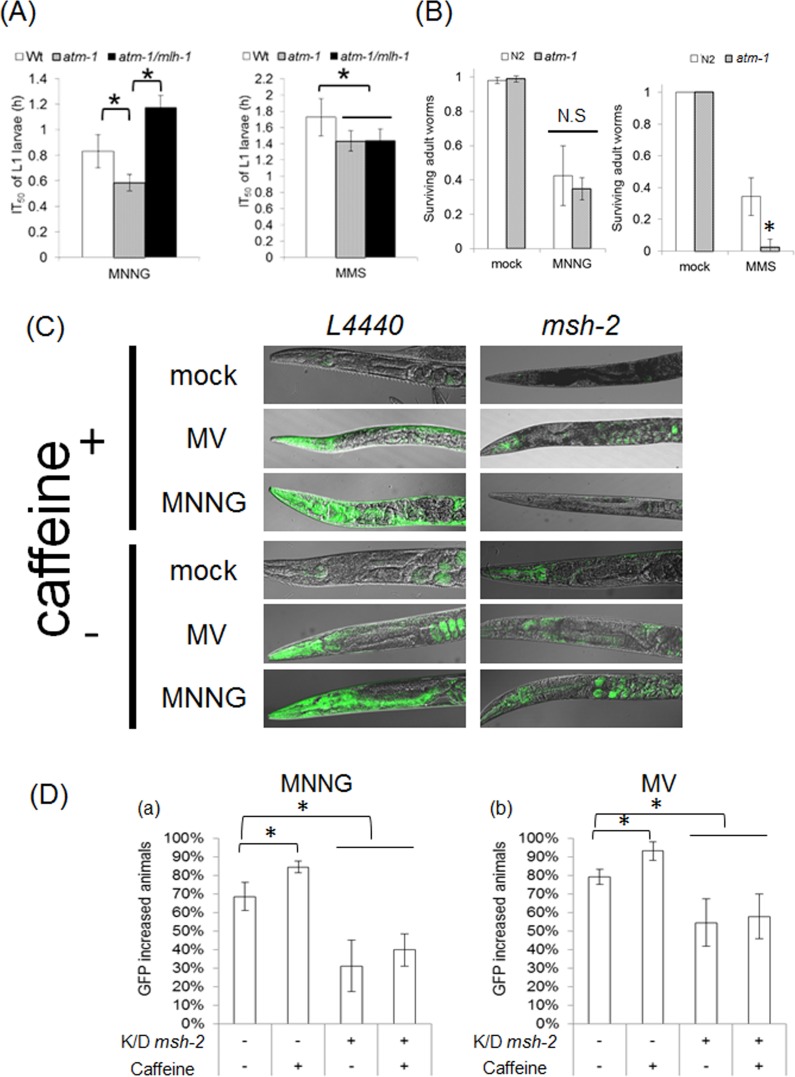
MMR induces autophagic cell death in an ATR-independent manner **(A)** IT^50^ of L1 larvae of N2, *atm-1(tm5027)* and *atm-1(tm5027)*/*mlh-1(ok1917)*. The synchronized starved L1 larvae of N2, *atm-1(tm5027)* and *atm-1(tm5027)*/*mlh-1(ok1917)* were treated with 0.6 mM MNNG or 0.05% MMS. After the treatments, worms were cultured for 4 days on NGM plates at 20°C. We then calculated IT_50_ or ID_50_ from the normalized percent growth (L1 to L4). IT_50_ was obtained from the logistic curve. Error bars show 95% confidence interval. * indicates *p*-value<0.05 by Student's *t*-test. **(B)** *atm-1(tm5027)* adult worms did not show sensitivity to MNNG. The adult worms of N2 and *atm-1(tm5027)* were treated with 3.4 mM MNNG or 0.5% MMS for 1 hour at 20°C. After the treatment, worms were cultured at 20°C. Twenty-four hours later, the survival rate was calculated. All data represent the mean ± S.D. from three independent experiments. * indicates p-value < 0.05 by Student's *t*-test. **(C)** Caffeine inhibition assay. Caffeine-treated (20 mM caffeine for at least 6 hours) *lgg-1* synchronized MAH236 (*lgg-1*p::GFP::*lgg-1*) adult worms cultured on control or RNAi plates were treated with 1.7 mM MNNG, 50 mM MV or 50 mM NaHSO_3_ for 7 hours at 20°C. After the treatments, worms were fixed with PFA. After washing two times with M9 buffer, worms were examined microscopically. **(D)** The fraction of animals with increased somatic GFP was measured. All data represent the mean ± S.D. from three independent experiments. * indicates *p*-value<0.05 by Student's *t*-test.

There are two hypotheses about how MMR induces cell death: “futile repair” and “signaling” [8]. In futile repair, repeated excision and resynthesis occur, and they lead to DSBs and genome instability [46]. It has been reported that MNNG generates DSBs in an MMR-dependent manner, and a DSB repair mutant shows sensitivity to MNNG [47,48]. Ataxia telangiectasia mutated (ATM) is a member of the PIKK family, and it is required for DSB repair, homologous recombination, non-homologous end-joining, and alternative end-joining [49]. In *C. elegans*, ATM-1, a homologue of ATM, also plays a critical role in DSB repair [50,51]. In order to analyze whether DSBs are generated by MNNG treatment, we performed drug resistance assays using *atm-1(tm5027)* worms. In *C. elegans*, *atm-1(tm5027)* L1 larvae showed sensitivity to both MNNG and MMS, but the sensitivity to MNNG was abrogated by deficiency of *mlh-1*, whereas the sensitivity to MMS was not (Supplementary Figure S7, Figure 6A). MNNG generates relatively stable DNA lesions such as O^6^-methylguanine, whereas MMS induces DSBs via accumulation of AP sites, which are converted to SSBs by AP endonucleases. From these facts, it is considered likely that MNNG induced DSBs in an MMR-dependent manner in dSCs, suggesting the presence of futile repair in dSCs. In contrast, adult worms did not show sensitivity to MNNG although they showed sensitivity to MMS (Figure 6B). This means that MNNG can not induce DSB in ndSCs, suggesting the absence of futile repair in ndSCs. Therefore, *C. elegans* MMR can induce cell death via both futile repair and signaling depending on the types of cell or stages.

During futile repair, in order to protect the template single-strand DNA, replication protein A (RPA) is accumulated on the template strand. This RPA recruits ATR, and the recruited ATR activates the signaling cascade [52]. This suggests that ATR may not be required for “signaling” cell death. Accordingly, we examined whether ATL-1 is responsible for ndSCs death. In *C. elegans*, the knockout of *atl-1* results in lethality. For this reason, we can not use an *atl-1* null mutant, although we can use an *atl-1* heterozygous mutant (*tm853*/*nT1*). This means that we could only decrease the *atl-1* expression level to only about 50% by either knockout or knockdown. In order to more efficiently inhibit ATL-1, we performed caffeine treatment. Caffeine is widely used as an inhibitor of ATM and ATR proteins, and in a previous study, treatment with 20 mM caffeine for at least 6 hours decreased the ATL-1 activity in *C. elegans* [53]. In our experiments, caffeine treatment did not inhibit expression of LGG-1, but rather appeared to slightly promote it under conditions of MNNG treatment (Figure 6C and D). These results suggest that ATL-1 is not responsible for autophagic cell death in ndSCs.

## DISCUSSION

In the present study, we demonstrated that *C. elegans* MMR induces autophagic cell death in response to DNA damage in non-dividing cells. Previous studies showed that MMR-induced cell death and cell cycle arrest are responsible for genome integrity and cancer prevention in mammalian dividing cells [4]. On the other hand, some reports showed that MMR factors are also present in non-dividing cells and MMR prevents carcinogenesis in non-dividing cells, such as kidney and liver [54]. Some reports showed DNA replication-independent functions of MMR, such as non-canonical MMR, which usually result in inaccurate DNA repair and induce mutations and cancers [18,19]. These facts are not in accord with clinical observations, and how MMR prevents cancers of non-dividing cells has remained unclear. Our finding that MMR induces cell death in non-dividing cells in response to DNA damage suggests one possible function of MMR in the prevention of carcinogenesis in non-dividing cells.

A recent study showed that 5-FU treatment induces autophagic cell death in an MMR- and AP endonuclease-dependent manner in *C. elegans* embryos, but other substrates of *C. elegans* MMR have not been identified [23]. In order to analyze the functions of *C. elegans* MMR, we first tried screening the substrates of *C. elegans* MMR using pachytene stage meiotic cells. These screenings showed that *mlh-1(ok1917)* pachytene stage meiotic cells have a deficiency of apoptosis induced by a wide range of DNA-damaging agents, such as MNNG and UVC (Figure 1). This suggests that *C. elegans* MMR, similarly to MMR in mammalian cultured cells, induces apoptosis in response to a wide range of DNA lesions, for example, O^6^-methylguanine, AP sites, and thymine dimers [9,14–17]. In contrast, *mlh-1(ok1917)* pachytene stage meiotic cells showed induction of normal levels of apoptosis by γ-rays irradiation (Figure 1G). While γ-rays irradiation generates reactive oxygen species (ROS), such as superoxide, γ-rays irradiation also directly induces DNA double-strand breaks [55]. As shown in Figure 1C, *mlh-1(ok1917)* pachytene stage meiotic cells can undergo induction of apoptosis in response to MV, which generates superoxide, indicating that *C. elegans* MMR responds to oxidative DNA damage caused by superoxide. Thus, γ-rays irradiation induced cell death not in response to oxidative lesions, but rather in response to more toxic DSBs.

In *C. elegans*, MMR factors are abundantly expressed in germ cells and embryos, while they are not as highly expressed in postembryonic somatic cells [40,41]. To examine whether *C. elegans* MMR works in postembryonic somatic cells, we measured the drug resistance during growth, and found that the development of *mlh-1(ok1917)* larvae showed higher IT or ID in response to some kinds of DNA-damaging agents compared to N2 larvae (Figures 2A and B). This means that *C. elegans* MMR induces cell death or cell cycle arrest in dividing somatic cells (dSCs), and deficiency of MMR results in DNA-damage-resistant growth of L1 larvae.

Next, we performed drug resistance assays using adult worms, and found that *mlh-1(ok1917)* adult worms are resistant to MNNG and MV treatments (Figures 3A and B). This suggests that *C. elegans* MMR induces non-dividing somatic cells' (ndSCs') death. In order to confirm whether MMR induces ndSCs' death, we performed AO staining. The results of AO staining suggested that *C. elegans* MMR induced cell death in response to the drug treatments in ndSCs such as those in pharynx, intestine and neurons (Figure 4). These results indicate that *C. elegans* MMR induces cell death independently of DNA replication.

In addition, we obtained interesting findings about the mechanism of cell death induced by MMR. In eukaryotic organisms, two models have been suggested for the mechanisms underlying MMR-induced cell death: “futile repair” and “signaling” [8]. In a previous study, it was shown that EXO1 is essential for inducing cell death by MMR in mammalian cultured cells, but the exonuclease activity of EXO1 is not required [10]. This suggests that MMR can induce cell death just by signaling, without futile repair. In our experiments, *atm-1(tm5027)* L1 larvae showed sensitivity to both MNNG and MMS (Figure 6A). On the other hand, *atm-1(tm5027)* adult worms did not show sensitivity to MNNG, whereas they were sensitive to MMS (Figure 6B). These results suggest that MNNG treatment generates DSBs in dSCs, but not in ndSCs. In addition, the sensitivity to MNNG in L1 larvae was abrogated by deficiency of MLH-1 (Figure 6A). Based on these facts, we hypothesize that MMR induces cell death by futile repair and/or signaling in dividing cells, and by only signaling in non-dividing cells.

In futile repair, RPA is accumulated on single-strand DNA, and RPA recruits ATR/ATRIP. Therefore, the fact that MMR can induce cell death by just signaling suggests that MMR can induce cell death independently of ATR/ATRIP in non-dividing cells. To examine the necessity for ATR/ATRIP in ndSCs' death, we performed caffeine-inhibition assays of the induction of LGG-1 using adult worms. The results of caffeine inhibition assays suggested that *C. elegans* MMR induced autophagic cell death independently of ATL-1, which means that *C. elegans* MMR induced autophagic cell death in ndSCs in an apparently different manner from that in embryos (Figures 6C and D) [23].

In HeLa cells, the MAPO1/AMPK pathway has been identified as a pathway that induces apoptosis in response to SN1-type alkylating agents in an MMR-dependent manner [56]. These factors are highly conserved among higher eukaryotes, and also in *C. elegans*. The fact that p53 is inactivated in HeLa cells implies that the MAPO1/AMPK pathway may also not require ATR/ATRIP. Furthermore, AMPK regulates autophagy via inhibition of mTOR, whose activity is not inhibited by caffeine, and indeed rather is promoted by it [57]. This is in accord with the results of our caffeine inhibition assay (Figure 6D), and implies that *C. elegans* MMR induces autophagic cell death via AMPK/mTOR in ndSCs.

As another possibly significant feature of autophagy induced by MMR, it is considered that MMR may prevent the accumulation of abnormal proteins. DNA lesions can induce not only replication errors but also transcription errors. Therefore, severe damage of genomic DNA leads to accumulation of abnormal proteins. MMR may contribute to the maintenance of cell function by surveying DNA lesions and inducing autophagy to degrade abnormal proteins.

We obtained the interesting findings that the sensitivity of *mlh-1(ok1917)* cells differed among pachytene stage meiotic cells, dSCs, and ndSCs (Figures 1-3). In addition, MMR induced autophagic cell death in different tissues in response to MNNG versus MV (Figures 4 and 5). In humans, MutSα has SQ/TQ sites, and the kinase inhibitor UCN-01 decreases the phosphorylation levels of MutSα and its binding affinity to O^6^-methylguanine [9]. Even though our findings might have been due simply to differences of drug penetration or just the difference of the presence or absence of DNA replication, which induces genome instability via replication stress, these differences may mean that MMR components may be appropriately modified depending on the growth stage or tissue in order to efficiently and appropriately protect each tissue. For example, *C. elegans* MMR can recognize more kinds of DNA damage in pachytene stage meiotic cells than in somatic cells, and this may be due to the various modifications of *C. elegans* MutSα in order to enable precise inheritance of the genomic information.

Recently much attention has been paid to dormant cancer cells. These cells are considered to be cancer stem cells and to show resistance to many anticancer agents. These cells are thought to be a cause of recurrent carcinoma because of the difficulty associated with killing them [58]. This resistance may be derived from differences in the DDR pathways between dividing and non-dividing cells. DDR pathways in non-dividing cells need to be examined in more detail. The results of the present study may represent a breakthrough for cancer therapy for dormant cancer stem cells.

## MATERIALS & METHODS

### Construction of a knockdown vector

The sequence information of H26D21.2 (Ce*msh-2*) was obtained from the NCBI database. PCR was conducted using two PCR primers listed in the Supplementary Table and KOD-Plus-polymerase (TOYOBO, Japan). The amplified products were digested with *Bgl* II and *Hind* III for cloning an approximately 1-Kbp fragment from the 5′ end. The digested products were then ligated to the L4440 vector digested with *BamH* I and *Hind* III.

### *C. elegans* strains and culture conditions

The wild-type strain (Bristol N2), RB1572[*mlh-1(ok1917)* III], JK2739[*lin-6*(*e1466*) *dpy-5*(*e61*) *I/hT2* [*bli-4*(*e937*) *let-?*(*q782*) *qIs48*] (*I;III*)], NL2511[*msh-6*(*pk2504*) I], MAH236; [*lgg-1*p::GFP::*lgg-1* + *odr-1*p::RFP]), and SS104 [*glp-4*(*bn2*)I] were supplied by the *Caenorhabditis* Genetics Center (Minneapolis, USA). *atm-1(tm5027)* mutant was supplied by the National BioResource Project (Tokyo, Japan). A deletion was verified in the *mlh-1* and *atm-1* genes by PCR using two primer pairs listed in the Supplementary Table. RB1572, NL2511 and *atm-1(tm5027)* mutant worms were backcrossed with Bristol N2 two times and maintained with the GFP balancer *hT2* to avoid the accumulation of mutations [31]. *lgg-1* reporter strain (*tm5027* [*lgg-1*p::GFP::*lgg-1* + *odr-1*p::RFP]), *atm-1(tm5027)*/*mlh-1(ok1917)* double mutant and balanced germline mutant [*mlh-1(ok1917) glp-4*(*bn2*) *lin-6*(*e1466*) *dpy-5*(*e61*) *I/hT2* [*bli-4*(*e937*) *let-?*(*q782*) *qIs48*] ], were generated by crossing each strain. Worms were cultured at 20°C on 50-mm NGM plates containing 0.3% (w/v) NaCl, 0.25% (w/v) polypeptone, 0.005% (w/v) cholesterol, 1 mM MgSO_4_, 1 mM CaCl_2_, 25 mM potassium phosphate (pH 6.0), and 0.17% (w/v) agar with a lawn of *Escherichia coli* (*E. coli*) OP50 [59].

### Synchronization of worms

Starved L1 larvae were prepared in order to obtain synchronized worms. Briefly, worms on NGM plates were harvested by washing out with distilled water and incubated in alkaline hypochlorite [500 mM NaOH and 1.2% (v/v) hypochlorite] until their bodies were completely dissolved (5-10 minutes). Eggs were then washed three times with S basal [50 mM potassium phosphate, (pH 6.0), containing 100 mM NaCl]. The eggs were hatched and synchronized by incubation at 20°C overnight without food [59].

### Measurement of lifespan

Synchronized L1 larvae were cultured on NGM plates until they developed to the adult stage (3 days). To determine lifespans, worms were harvested with M9 buffer (22 mM KH_2_PO_4_, 42 mM Na_2_HPO_4_, l mM MgSO_4_, 85 mM NaCl) and transferred to 50-mm NGM plates containing 10 μg/ml 5-fluoro-2′-deoxyuridine with a lawn of *E. coli* OP50. Worms that failed to move spontaneously or respond to touch were counted as dead. Worms were counted every 2 days [60].

### L1 growth assay

The time-course drug treatments were performed using synchronized L1 worms in M9 buffer containing various drugs at 20°C. Then, worms were transferred to NGM plates and incubated for 4 days. UVC irradiation was performed to synchronized L1 worms, and then worms were incubated for 4 days. After 4 days, the percentage of worms that grew from L1 to adults was calculated, and the half-maximal inhibitory time or dose was obtained by the three-parameter logistic curve using R [55]. At least 200 animals were counted in each condition.

### Quantification of germ line apoptosis

Synchronized L1 larvae were cultured on NGM plates until they developed to the young adult stage (2 days). They were harvested with M9 buffer and then treated with various DNA-damaging agents in M9 buffer at 20°C for 24 hours. Worms were then immobilized in M9 buffer containing 20 mM sodium azide and observed using a differential interference contrast (DIC) microscope [33]. At least 150 gonad arms were counted in each condition.

### Adult worm drug resistance assay

Synchronized L1 larvae were cultured on NGM plates until they completely developed to the adult stage (4 days). They were harvested with M9 buffer and then treated with various DNA-damaging agents in M9 buffer for 1 hour at 20°C. They were transferred to NGM plates and incubated for 1 day, and the survival rate was then calculated. At least 200 animals were counted in each condition.

### AO staining

Synchronized L1 larvae were cultured on NGM plates until they developed completely to the adult stage (4 days). Adult worms were harvested with M9 buffer, and treated with various DNA-damaging agents in M9 buffer for 1 hour at 20°C. Worms were then washed with M9 buffer two times and stained with 5 mg/ml acridine orange (AO) in M9 buffer for 5 minutes. Worms were destained two times with 1 ml of M9 buffer for 10 min, and fixed with phosphate buffered saline (pH 7.4) (PBS) containing 4% paraformaldehyde (PFA). After washing two times with 1ml of M9 buffer, they were observed using fluorescence microscopy with excitation by a 488 nm argon laser [42]. At least 150 animals were counted in each condition.

### Feeding RNAi

Knockdown analysis was performed using standard feeding methods [61,62]. *E. coli* HT115 (DE3) was transformed with L4440 vectors and cultured overnight in Luria-Bertani (LB) medium containing 25 μg/ml carbenicillin (Cb), 15 μg/ml tetracycline (Tet), and 320 μM isopropyl β-D-1-thiogalactopyranoside (IPTG) at 30°C. After additional supplementation with Cb, Tet, and IPTG (final 50 μg/ml, 30 μg/ml, and 640 μM, respectively), overnight cultures were seeded on NGM plates (RNAi plates). Worms were primarily cultured on RNAi plates for 3 days. Eggs were then harvested using the alkaline bleach method. The eggs were transferred to fresh RNAi plates and allowed to develop completely to the adult stage (4 days). To confirm the effects of RNAi, reverse transcription-polymerase chain reaction (RT-PCR) was carried out using the primer pairs listed in the Supplementary Table and GoTaq (Promega, USA).

### Reporter assay

MAH236 worms cultured on RNAi plates were harvested with M9 buffer and treated with DNA-damaging agents for 7 hours at 20°C in M9 buffer. Worms were immediately fixed with PBS containing 4% PFA for 10 minutes at 20°C. After washing two times with M9 buffer, worms were observed using fluorescent microscopy with excitation by a 488 nm argon laser [23]. At least 150 animals were counted in each condition.

### Semi-quantitative RT-PCR

In order to determine the expression levels, semi-quantitative RT-PCR was carried out using the primer pairs listed in the Supplementary Table.

### Microscopy

DIC microscopy and fluorescence microscopy were performed using a Carl Zeiss LSM510 microscope (Carl Zeiss, Germany).

### Statistics

Qualitative data were representative data of at least three experiments. Unless otherwise noted, quantitative data were expressed as the mean ± S.D. The significance of differences was examined using Student's *t*-test or the Mann–Whitney U-test. *p*-values<0.05 were considered significant.

## SUPPLEMENTARY TABLE AND FIGURES


