# Supplementary material for: A novel DNA damage response mediated by DNA mismatch repair in Caenorhabditis elegans: induction of programmed autophagic cell death in non-dividing cells
Source: Genes Cancer. 2015 Jul;6(7-8):341–55. doi: 10.18632/genesandcancer.70 (PMC4575921; doi:10.18632/genesandcancer.70)
Supplement: Supplementary file 1 [file ganc-06-341-s001.pdf]

Cloning		
msh-2	forword	5’-GAAGATCTATGAGTGGAGGAAAAGACGAAGCCA-3’
	reverse	5’-ATGCGGCCGCTTATTTGACAAGGCTGAGAATGGCT-3’

Verification of deletion		
mlh-1(External)	Forward	5’-CACATCGCTCGAAGACTTTGTACAA-3’
	Reverse	5’-CTGGTGGCCTACCCACCAAACTCAT-3’
mlh-1(Internal)	Forward	5’-GTCCGCCATTCTCACTAAATCTCGA-3’
	Reverse	5’-CTGGTGGCCTACCCACCAAACTCAT -3’
atm-1(External)	Forward	5’-CGGAAAAACGATGTACCGATGGCCA-3’
	Reverse	5’-AGGGACCTGCGTCTCTCTTCGCCAC-3’
atm-1(Internal)	Forward	5’-CGGAAAAACGATGTACCGATGGCCA-3’
	Reverse	5’-CCGAAGCATCGCCTTCTCCAACATA-3’
msh-6(External)	Forward	5’-TTATCGAAAGACCGGAAACCAAAA-3’
	Reverse	5’-GAAGCCCTTCGACGAGATTTCAGC-3’
msh-6(Internal)	Forward	5’-CCGACCCCTTGATCGAAACGG-3’
	Reverse	5’-CTGGTGGCCTACCCACCAAACTCAT -3’

Semi-quantitative PCR		
ama-1	forward	5’-TTCCAAGCGCCGCTGCGCATTGTCTC-3’
	reverse	5’-CAGAATTTCCAGCACTCGAGGAGCGGA-3’
tbg-1	forward	5’-TGATGACTGTCCACGTTGGA-3’
	reverse	5’-CGTCATCAGCCTGGTAGAACA-3’
msh-2	forward	5’-GAAGAAAAGTCGATCCGCAA-3’
	reverse	5’-CAAGAGGAAGAAGATTCGGTC-3’
egl-1	forward	5’-ATGCTGATGCTCACCTTTGCC-3’
	reverse	5’-GATGGAAGAGGCTTCTGTCGG-3’
vps-34	forward	5’-CGGGATCCCAAACCAATGCC-3’
	reverse	5’-GTGAATAATATCAGAAATCATAGCC-3’

Supplementary Table. The primers used in this study. These primers were used for the cloning of *msh-2* for knockdown, Verification of deletion of mutants and semi-quantitative PCR.

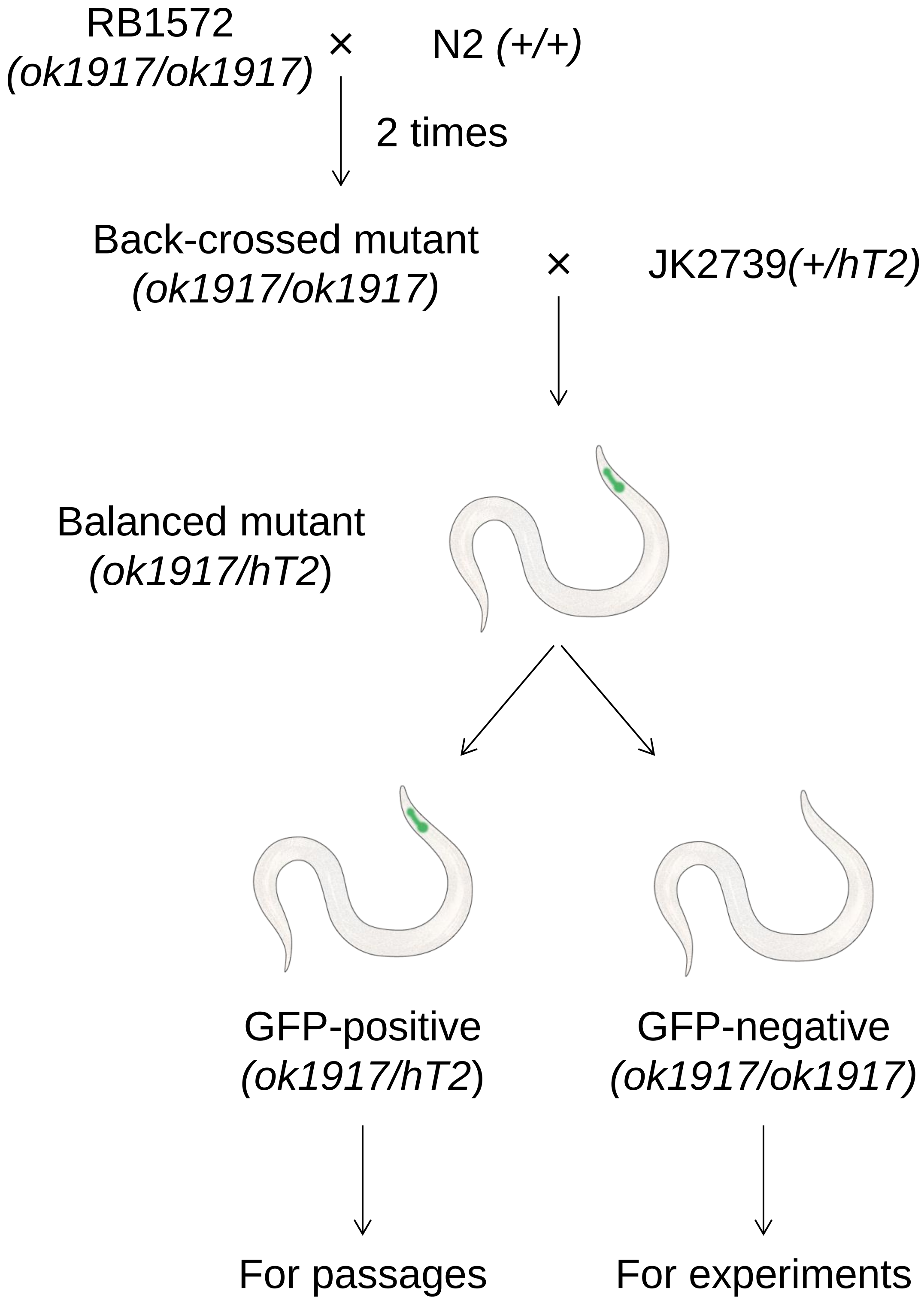

Supplementary Figure S1. Stable maintenance of the MMR mutant. In order to analyze under stable genetic conditions, we used the balancer chromosome *hT2* inserted into *myo-2*p::GFP. Backcrossed *mlh-1*(*ok1917*) worms were maintained with *hT2* and GFP-negative worms were isolated for experiments. A photograph of a worm was obtained from the Togo picture gallery (http://g86.dbcls.jp/~togoriv/). ©2011 DBCLS Licensed under a Creative Commons Attribution 2.1 Japan License.

(A)

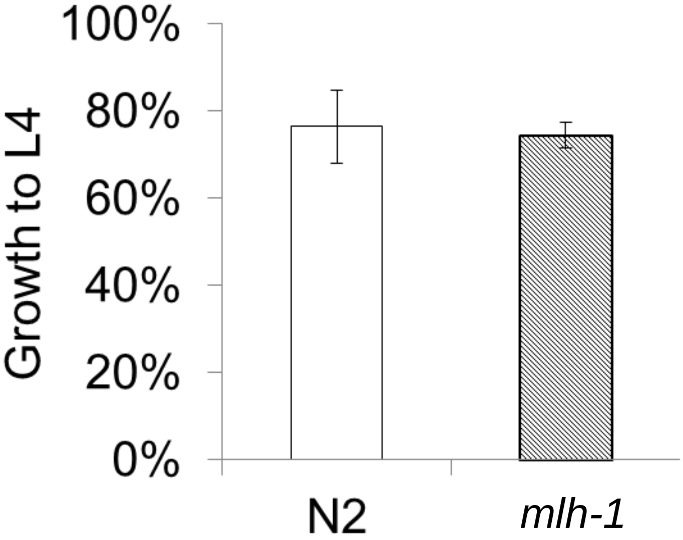

(B)

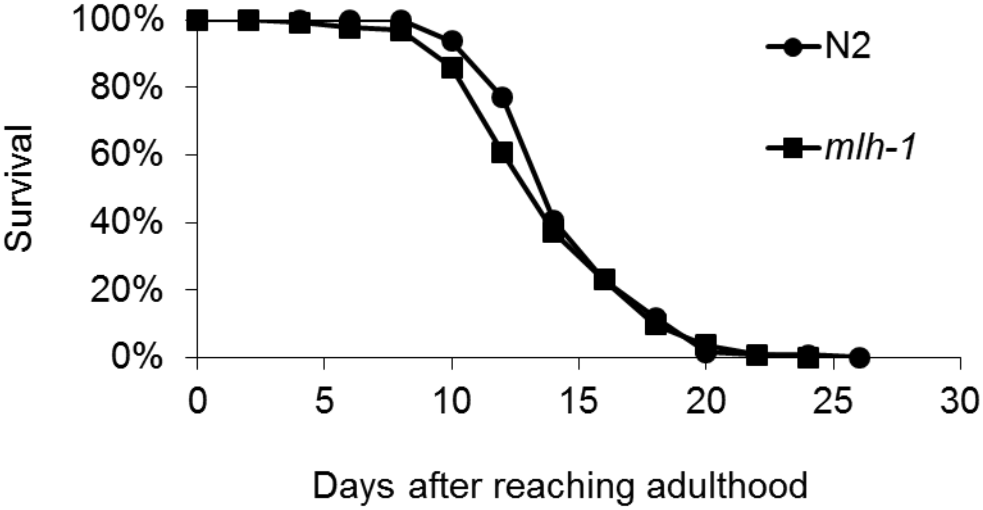

Supplementary Figure S2. Basic phenotypes of *mlh-1*(*ok1917*) worms. (A) Percent growth (L1 to adult). Data represent the mean ± S.D. from three independent experiments. No significant difference was observed (*p*-value>0.05 by Student's *t*-test). (B) The lifespan survival curve. ● and ■ represent N2 (n=126) and backcrossed *mlh-1*(*ok1917*) (n=135), respectively. Data represent the results from three independent experiments. No significant difference was observed in mean or 50% lifespan (*p*-value>0.05 by Student's *t*-test).

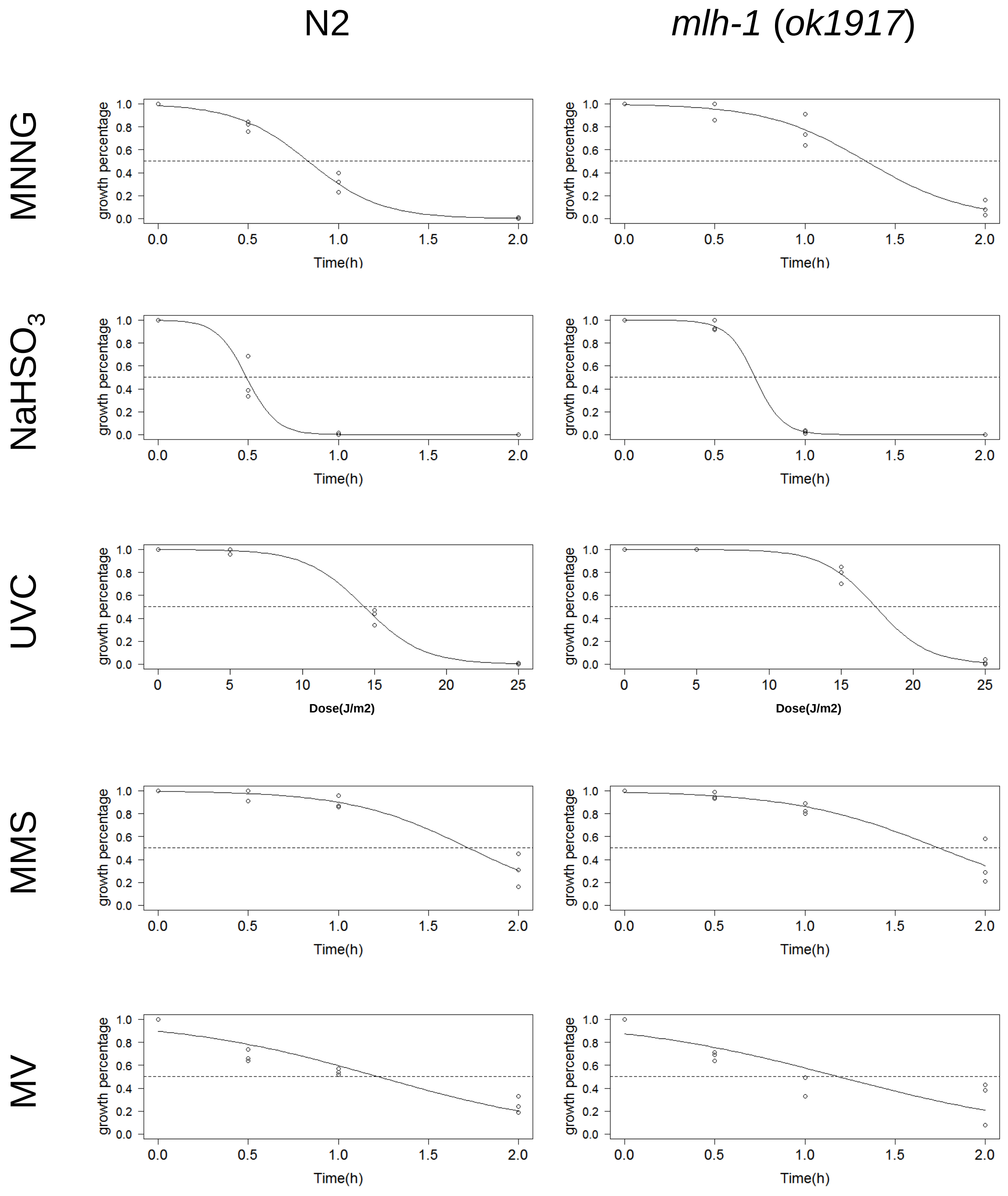

Supplementary Figure S3. The logistic curve of L1 growth assays. The synchronized starved L1 larvae of N2 and *mlh-1*(*ok1917*) were subjected to time-course drug or irradiation treatments with 0.6 mM MNNG, 60 mM $NaHSO_3$, 40 mM MV, 0.05% MMS or UVC irradiation. After the treatments or irradiation, worms were cultured for 4 days on NGM plates at 20 ^{o}C. Then, the normalized percent growth (L1 to L4) was measured. The time- and dose-response curves were obtained with three-parameter logistic curves using R. The dotted lines represent 50% line.

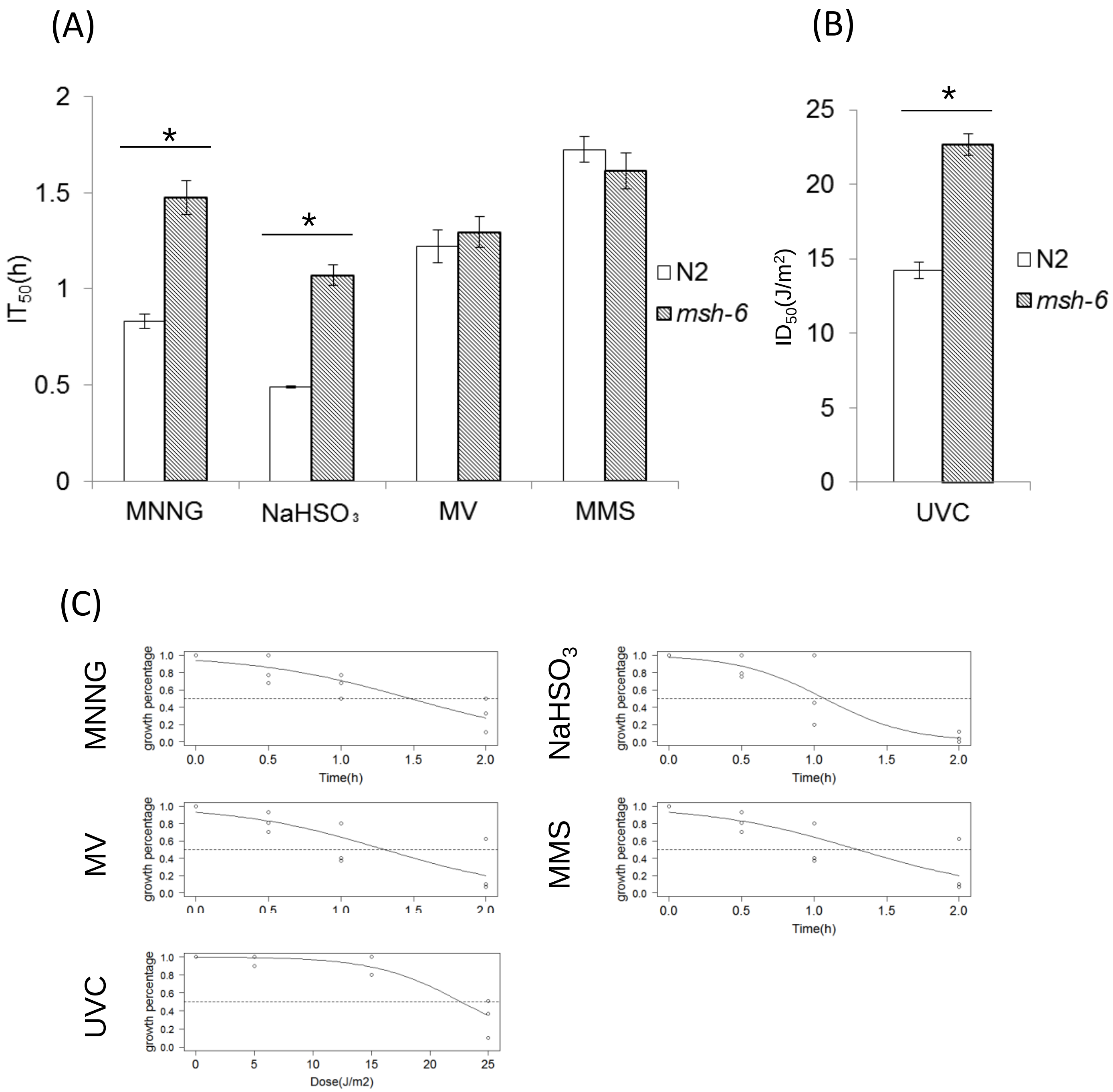

Supplementary Figure S4. L1 growth assay of *msh-6* (*pk2504*) worms. (A) The synchronized starved L1 larvae of N2 and *msh-6* (*pk2504*) were treated with (A) 0.6 mM MNNG, 60 mM NaHSO3, 40 mM MV, or 0.05% MMS and (B) irradiated with UVC. After the treatments or irradiation, worms were cultured for 4 days on NGM plates at 20 ^{o}C. We then calculated IT50 or ID50 from the normalized percent growth (L1 to L4). Error bars show 95% confidence interval. * means p-value<0.05 by Student's t-test. (C)The logistic curve of L1 growth assays. The time- and dose-response curves were obtained with three-parameter logistic curves using R. The dotted lines represent 50% line.

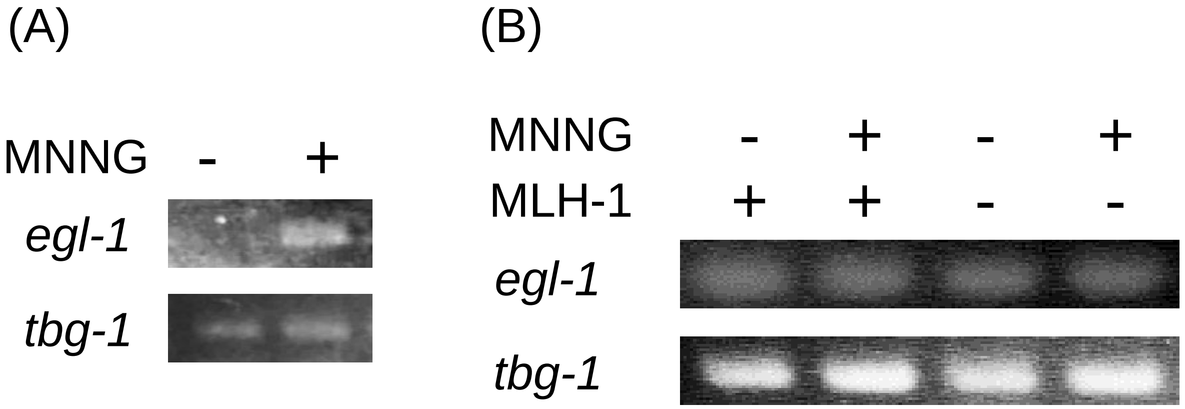

Supplementary Figure S5. The expression levels of *egl-1*. (A) MNNG treatment induces *egl-1* expression in N2 adult worms. The N2 adult worms were treated with 3.4 mM MNNG for 1 hour at 20°C. Then, total RNA was purified and reverse transcribed. The expression levels of *egl-1* were determined by semi-quantitative PCR. *tbg-1* was used as the loading control. (B) The adult worms of wild-type or *ok1917* germline deficient worms (*glp-4*(*bn2*)) were treated with 3.4 mM MNNG for 1 hour at 20°C. Then, total RNA was purified and reverse transcribed. The expression levels of *egl-1* were determined by semi-quantitative PCR. *tbg-1* was used as the loading control.

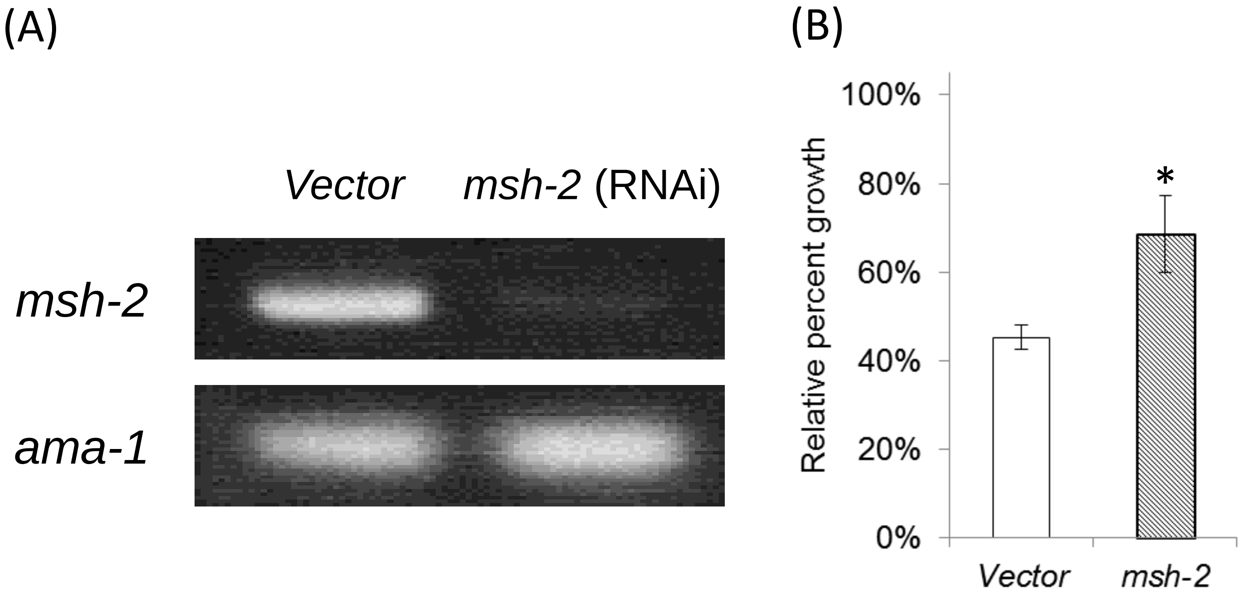

Figure S6. Confirmation of knockdown of the *msh-2*-gene by semi-quantitative PCR. (A) The knockdown analysis was performed using standard feeding methods. Worms were cultured on RNAi plates for 4 days from the egg stage, and total RNA was then prepared. The decrease of mRNA level was analyzed by semi-quantutative PCR. Considering the difference of rate of germ cells between vector and *msh-2* (RNAi), *ama-1* was used as the loading control. (B) *msh-2* knockdown induces the resistance to MNNG. The synchronized L1 larvae of vector or *msh-2* RNAi were treated with 0.6 mM MNNG for 1 hour at 20 ^{o}C. After the treatment, worms were cultured at 20 ^{o}C for 4 days. We then calculated the normalized percent growth (L1 to L4). The data represents the mean ± S.D. from three independent experiments. * means *p*-value<0.05 by Student's *t*-test.

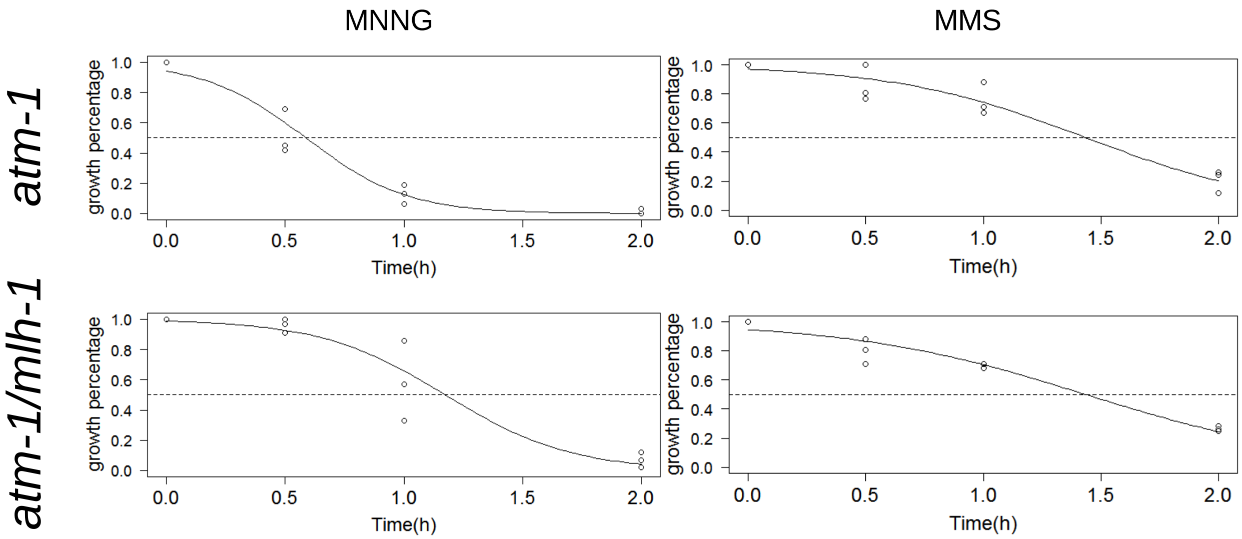

Figure S7. MNNG induces DSB in *C. elegans* dividing cells. The logistic curve of L1 growth assays of *atm-1*(*tm5027*) and *atm-1*(*tm5027*)/*mlh-1*(*ok1917*) worms. The synchronized starved L1 larvae were subjected to time-course drug treatment with 0.6 mM MNNG or 0.05% MMS. After the treatment, worms were cultured for 4 days on NGM plates at 20°C. Then, the normalized percent growth (L1 to L4) was measured. The time-response curve was obtained with a three-parameter logistic curve using R. The dotted lines represent 50% line.
